# Onions in Intermittent Fever

**Published:** 1867-03

**Authors:** A. Given

**Affiliations:** Louisville, K’y.


### ONIONS IN INTERMITTENT FEVER.

LOUISVILLE, K'y., *Feb. 14, 1867.*

DR. N. S. DAVIS:—

*Dear Sir:*—My attention has been called to a simple, and supposed effectual, remedy for arresting the chills of miasmatic origin. I was consulted, last summer, by Mr. H., for chills; he had had them for several weeks. I gave him a prescription, which soon controlled the disease for a short time; after which the chills returned at regular intervals. He being averse to taking medicine, wished to try a simple remedy before taking any more from me. When he felt the chill coming on, he went to his room, undressed, sliced a large onion, rubbed his chest with the pieces, and then went to bed, covered up warm, and was soon in a perspiration, having no chill nor fever. He repeated the operation as often as he felt the chill approaching, and it checked the paroxysm every time, and removed them entirely after the third application. I gave the subject but little attention, thinking it was a spontaneous cure.

Yesterday, my attention was again called to the subject, by a gentleman of undoubted veracity. He informs me that he has broken up the chills on two of his children. One case had resisted all the treatment given by a physician, after which the onions were tried. They were beaten to a pulp and bound on the wrists and soles of the feet, one hour before the time of the expected chill. In one case, the chills never returned after the first application; in the other, it returned in fourteen days, when the onion was again applied; since then, the chill has not returned. He also informed me that he had tried the onion in seven other cases, with the same result.

It seems to me that this is a subject worthy of investigation, simple though it be. Here are ten corroborative cases, the paroxysm in each having been arrested in the cold stage, and the periodicity broken up in all. It will be seen that the mode of application was different: in my case, the juice was applied to the chest; in the others, the onion was applied to the extremities, yet the result was the same. It is hardly probable that the onion would be applied in all the ten cases at that time when nature was about to free the system of the offending difficulty, independent of remedial agents. If not, then the onion is the cheapest and best antiperiodic of the materia medica.

I have called your attention to this subject, not knowing whether it has been investigated by the profession, or not. I have used the onion for the last seven years, in the early stages of infantile pneumonia, with the happiest effects. I was called to see a child last night. I found it breathing short and laborious; uneasy and restless; crying or groaning at every breath; with a hacking cough. In auscultating, I found a crepitus over a portion of the right lung. I had onions pounded fine, and covered the right side of the chest with them. They were quieting and soothing, and the child was asleep in half an hour, and breathing comparatively easy. This morning the child is doing well. I do not know where I got the suggestion for the use of the onion in these cases, except from some of your lectures.

I hope you will pardon me for encroaching upon your time, to ask you to read a letter on what may be to you a familiar and exploded subject. Yet, I am sure that I have never tried any remedy, in the early stages of pneumonia of children, that has given me as good and immediate results as the onion. Hoping that the onion may prove to be as effectual in intermittents as its advocates claim, I remain, Yours, truly,

A. GIVEN.

---

Reopening of the Knoxville, Tenn., Deaf and Dumb Asylum.—The Knoxville (Tenn.) Asylum for the Deaf and Dumb has been reopened after a five years' suspension. It was occupied during the late war as a hospital by both contending forces in succession. As the "Asylum U. S. General Hospital No. 1," it achieved no little reputation during the campaign in East Tennessee.

